# Osteopetrorickets and calcium homeostasis in children with osteopetrosis: an endocrinological single-center study

**DOI:** 10.3389/fendo.2026.1854949

**Published:** 2026-08-13

**Authors:** Ebru Gök, Emre Sarıkaya, Leyla Kara, Uğur Berber, Ülkü Gül Şiraz, Alper Özcan, Ebru Yılmaz, Ekrem Ünal, Musa Karakükcü, Nihal Hatipoğlu

**Affiliations:** 1 Division of Pediatric Endocrinology, Department of Pediatrics, Erciyes University, School of Health Sciences, Kayseri, Türkiye; 2 Division of Pediatric Hematology and Oncology, Department of Pediatrics, Erciyes University, School of Health Sciences, Kayseri, Türkiye; 3 Medical Point Hospital, Pediatric Hematology and Oncology Clinic, Gaziantep, Türkiye; 4 Department of Nursing, School of Health Sciences, Hasan Kalyoncu University, Gaziantep, Türkiye

**Keywords:** bone metabolism, calcium homeostasis, hematopoietic stem cell transplantation, hypercalcemia, osteopetrorickets, osteopetrosis, pediatric endocrinology, TCIRG1

## Abstract

**Background:**

Osteopetrosis is a rare inherited disorder caused by impaired osteoclast number or function, leading to increased bone density, fractures, neurologic complications, and disturbances in calcium-phosphate homeostasis. This study aimed to describe the endocrine manifestations of childhood osteopetrosis, particularly osteopetrorickets, and to evaluate treatment responses and post-transplant calcium disorders.

**Methods:**

We retrospectively reviewed 17 children diagnosed with osteopetrosis at a single tertiary center between 2015 and 2025. Clinical, biochemical, radiologic, genetic, and treatment data were analyzed.

**Results:**

The median age at diagnosis was 14 months (range, 15 days–130 months), and short stature was observed in 13 of 17 patients (76.4%). Ophthalmologic abnormalities were present in 10 patients (58.8%), hearing loss in 7 patients (41.1%), and hepatosplenomegaly in 7 patients (41.1%). *TCIRG1* was the most frequent mutation, followed by *CLCN7, TNFSF11, TNFRSF11A*, and *CA2*. Osteopetrorickets was identified in 13 of 17 patients (76.4%); among these patients, hypocalcemia occurred in 11 of 13, hypophosphatemia in 8 of 13, and vitamin D deficiency in 4 of 13. Generalized osteosclerosis was observed in all patients, whereas classic osteopetrosis-associated radiographic findings, including bone-in-bone appearance, sandwich vertebrae, and Erlenmeyer flask deformity, were identified only in a subset of patients. Hematopoietic stem cell transplantation was performed in 11 of 13 patients with osteopetrorickets. Four of these 11 patients died during the early post-transplant period. Among patients with osteopetrorickets who underwent hematopoietic stem cell transplantation (HSCT) (n=11), seven surviving patients achieved complete resolution of rickets (7/11, 63.6%), allowing discontinuation of replacement therapy within 15 days to 8 months. Post-transplant hypercalcemia developed in 4 of 13 transplant recipients (30.8%).

**Conclusions:**

Osteopetrorickets is a frequent and clinically significant complication of pediatric osteopetrosis. Early recognition of mineral disturbances, genotype-based treatment planning, and close surveillance for rebound hypercalcemia after transplantation are essential to improve outcomes.

## 1 Introduction

Osteopetrosis (marble bone disease) is a rare, hereditary bone disorder characterized by reduced bone resorption due to a deficiency in the number or function of osteoclasts. Increased bone density can lead to various clinical manifestations, including fractures, bone marrow failure, neurological complications, and disturbances in mineral metabolism (1, 2). Inheritance patterns may include autosomal recessive (ARO), autosomal dominant (ADO), or X-linked inheritance (1). The most severe form is known as infantile malignant osteopetrosis (IMO) (3). In 70% of IMO cases, mutations are found in T-cell immune regulator 1 (*TCIRG1*), Chloride channel 7 (*CLCN7*), Osteopetrosis-associated transmembrane protein 1 (*OSTM1*), Sorting Nexin 10 (*SNX10*), Pleckstrin Homology Domain-Containing Family M Member 1 (*PLEKHM1*), Receptor Activator of Nuclear Factor Kappa-B Ligand (*RANKL*), and Carbonic Anhydrase 2 (*CA2*) (4). The most common mutations are in *TCIRG1* and *CLCN7*; over 50% of severe ARO cases are caused by *TCIRG1* mutations (5–7). Genotype–phenotype correlations are clinically relevant because they may affect disease severity, neurological involvement, and treatment eligibility; notably, *CLCN7* mutations may be associated with neurodegenerative manifestations, for which hematopoietic stem cell transplantation is generally contraindicated (8).

Clinically, IMO presents growth retardation, macrocephaly and frontal bossing, short stature, osteomyelitis, hypocalcemia, convulsions, and neurological deficits that are typically secondary to nerve compression. Impaired bone resorption can lead to narrowing of the bone marrow cavities and may result in death within the first ten years of life (2, 9–13). Radiologically, findings characteristic of osteopetrosis, such as “bone-in-bone,” “sandwich vertebra,” and “Erlenmeyer flask” deformities, may be observed (1, 13).

In patients with osteopetrosis, impaired calcium mobilization due to abnormal osteoclast activity can lead to secondary hyperparathyroidism from hypocalcemia, resulting in a paradoxical condition known as “osteopetrosis-rickets” (14). While calcium and vitamin D supplementation are recommended for the treatment of this condition, high-dose calcitriol has been reported to increase bone mass (15, 16). Osteopetrorickets is clinically important because it may be overlooked in the presence of diffusely increased bone density, although impaired mineralization may coexist with osteosclerosis. Its diagnosis requires careful integration of biochemical abnormalities, including hypocalcemia, hypophosphatemia, elevated alkaline phosphatase, and/or secondary hyperparathyroidism, with radiological evidence of rickets. Following HSCT, restoration of osteoclast function may induce rapid skeletal remodeling and calcium mobilization from bone, thereby predisposing patients to post-transplant hypercalcemia. However, data on the frequency, timing, severity, and management of calcium disturbances after HSCT in children with osteopetrosis remain limited. Similarly, the relationships among genotype, osteopetrorickets, treatment response, and post-transplant calcium homeostasis have not been fully defined. Therefore, evaluating these endocrine complications in a pediatric cohort may provide clinically relevant information for diagnosis, monitoring, and post-transplant management. The only curative option for IMO is HSCT. When performed early, serious complications can be prevented (3, 9). However, transplant outcomes vary according to genotype, disease severity, age at transplantation, donor availability, and pre-existing neurological involvement, and are generally poor in cases of *CLCN7* and *CA2* mutations (17–20). Therefore, treatment-related outcomes should be interpreted in the context of genotype–phenotype associations and baseline clinical status.

This study aimed to evaluate the frequency and clinical characteristics of osteopetrorickets in children with osteopetrosis, to describe associated biochemical and radiological abnormalities, and to assess treatment responses and post-transplant calcium disturbances in relation to the clinical course and underlying genetic background.

## 2 Materials and methods

### 2.1 Study design and patient selection

This retrospective study includes all patients diagnosed with osteopetrosis who presented to the Departments of Pediatric Endocrinology and Pediatric Hematology and Oncology at Erciyes University between 2015 and 2025. Patients who received a diagnosis of osteopetrosis based on clinical and radiological findings and who had completed genetic analysis were included in the study.

The diagnosis of osteopetrosis was primarily based on characteristic radiographic findings, including generalized osteosclerosis, bone-in-bone appearance, sandwich vertebrae, Erlenmeyer flask deformity, and skull-base sclerosis, supported by clinical features and confirmed by molecular genetic testing.

### 2.2 Clinical evaluation

A detailed medical history, including the patient’s family history, symptoms, and fracture history, was obtained. Anthropometric measurements, including height and weight, were obtained, and an abdominal examination was performed to assess for hepatosplenomegaly, along with a neurological examination to assess for cranial nerve disorders. The respective departments evaluated visual and hearing examinations.

### 2.3 Laboratory and radiological evaluation

At the time of presentation, serum calcium (Ca), phosphorus (P), alkaline phosphatase (ALP), parathyroid hormone (PTH), and 25-hydroxyvitamin D levels were measured, and a complete blood count was performed to assess for possible cytopenias. Although normal ranges vary with age, the lower and upper limits for serum calcium were set at 8.5 and 10.5 mg/dL, respectively, and a serum phosphorus level below 3.1 mg/dL was considered hypophosphatemia. A serum 25-hydroxyvitamin D level below 12 ng/ml was considered vitamin D deficiency, a level between 12 and 20 ng/ml was considered vitamin D insufficiency, and a level above 20 ng/ml was considered normal. Radiological evaluation was based on conventional radiographs, and skeletal findings related to osteopetrosis and rickets were recorded. Patients with calcium, phosphorus, and vitamin D deficiencies and/or radiological findings such as metaphyseal irregularity and brush-like appearance were diagnosed with osteopetrorickets. Patients with hypocalcemia and/or hypophosphatemia, or those with radiological findings of rickets (osteopetrosis), were given calcium, calcitriol, and/or phosphorus supplementation.

### 2.4 Genetic analysis and HSCT-related treatment

Molecular genetic analyses of the cases included the *TCIRG1, CLCN7, TNFRSF11A, TNFSF11, OSTM1, CA2, NEMO*, and *CTSK* genes, which were studied using NGS methods at various centers. When evaluated in conjunction with the patients’ family segregation patterns and clinical presentations, these gene mutations were found to be associated with the conditions. Treatment decisions were made through multidisciplinary evaluation, considering the clinical phenotype, disease severity, neurological involvement, genetic background, donor availability, and expected benefit of transplantation. HSCT was considered primarily in patients with severe osteopetrosis caused by osteoclast-intrinsic defects when the clinical phenotype suggested potential benefit. *CLCN7*-related osteopetrosis was considered a heterogeneous entity, and HSCT was reserved for selected cases based on disease severity and neurological status. HSCT was not considered curative in osteoblast-lineage defects, such as *TNFSF11*-related RANKL deficiency.

A total of 17 hematopoietic stem cell transplantation procedures were performed in 13 patients diagnosed with infantile osteopetrosis, including repeated transplantations performed because of graft failure. Of these transplantations, 3 were performed from human leukocyte antigen-matched sibling donors, 4 from matched unrelated donors, 2 from matched family donors, and 8 from mismatched related donors. Among the 13 transplanted patients, the most frequently used conditioning regimen was busulfan and fludarabine, administered to 6 patients (46.1%). For serotherapy, rabbit anti-thymocyte globulin was administered at 10–30 mg/kg, depending on donor type. For graft-versus-host disease prophylaxis, cyclosporine was combined with either low-dose methotrexate or mycophenolate mofetil, depending on donor type. In patients who underwent haploidentical transplantation, rituximab was administered prophylactically at a dose of 200 mg/m² before transplantation.

### 2.5 Statistical analysis

Statistical analyses were performed using descriptive methods because of the small sample size and retrospective design. Continuous variables were presented as median and range, and categorical variables were presented as numbers and percentages. Basic comparisons were performed between patients with and without osteopetrorickets and between transplanted patients with and without post-transplant hypercalcemia. Categorical variables were compared using Fisher’s exact test, and continuous variables were compared using the Mann–Whitney U test where appropriate. Because of the limited sample size, the results of comparative analyses were interpreted cautiously.

This study was approved by the Erciyes University Clinical Research Ethics Committee under decision number 2023/146 dated March 8, 2023.

## 3 Results

### 3.1 Patient clinical characteristics and neurological findings

A total of 17 patients diagnosed with osteopetrosis were included in the study, comprising 12 females and 5 males. The median age at diagnosis was 14 months (15 days–130 months). Five of the cases (29.4%) had a family history of the condition (**Table 1**).

**Table 1:** Anthropometric and clinical characteristics of the patients.

Patient no.	Age	Gender	Weight (kg)	Weight (SDS)	Height (cm)	Height (SDS)	BMI (kg/m^2^)	BMI (SDS)	FH	HSM	OF	NF
1	32 d	F	2,990	-2,19	47,5	-2,55	12,85	-1,37	+	–	N	–
2	17 mo	M	9,2	-1,82	73,5	-2,69	17	0,25	–	–	VL	–
3	13 mo	F	7,9	-1,69	66,5	-3,53	17,9	0,87	–	–	N	–
4	34 mo	F	12,3	-1,01	80,5	-3,51	19	2,14	–	–	VL	–
5	9 mo	M	7,7	-1,47	66	-2,4	17,7	0,1	+	–	N	FP
6	16 mo	F	9,4	-0,83	74,5	-1,76	16,9	0,44	–	+	OA	–
7	15 mo	F	7,6	-2,3	74	-1,47	13,9	-2,22	–	+	NS	–
8	2 mo	M	3,6	-2,69	50	-3,23	14,4	-1,34	+	–	N	–
9	8 mo	F	8	-0,09	62	-2,69	20,8	2,19	–	+	OA	–
10	13 d	F	3	-1,35	49	-0,9	12,49	-1,14	+	+	N	–
11	14 mo	F	10,6	0,7	80	1,1	16,6	0,02	–	–	N	–
12	53 mo	F	13	-2,11	94	-2,68	14,7	-0,53	–	+	NS	–
13	71 mo	F	13	-3,34	88	-5,74	16,8	0,7	+	–	OA	–
14	4 mo	M	5	-2,13	55	-3,09	16,53	-0,36	–	+	OA	–
15	16 mo	F	7,5	-2,63	69	-3,53	15,8	-0,43	–	+	N	–
16	130 mo	F	30	-1,22	129,2	-2,37	18	-0,01	–	–	NS	–
17	3 mo	M	3,6	-2,89	53	-2,44	12,8	-2,56	–	–	NS	–

M, male; F, female; d, days; mo, months; BMI, body mass index; SDS, standard deviation score; FH, family history; HSM, hepatosplenomegaly; OF, ocular finding; NF, neurological finding; VL, vision loss; OA, optic atrophy; NS, nystagmus; FP, facial paralys.

The most common presenting complaints were, in order: fever (n=4, 23.5%), vision problems (n=3, 17.6%), feeding difficulties (n=3, 17.6%), leg deformities (n=1, 5.8%), and fractures (n=2, 11.7%). 23.5% of the cases (n=4) were asymptomatic and received a diagnosis following screening due to a family history (abnormalities in blood counts or calcium metabolism, findings on plain radiographs, or genetic test results consistent with osteopetrosis) (**Table 2**).

**Table 2A:** Genotype–phenotype characteristics of patients with osteopetrosis.

Genetic mutation (n, %)	Common cinical findings	Radiological features	Presence of osteopetrorickets (n)
TCIRG1 (n=9, 52.9%)	Short stature, hepatosplenomegaly, optic atrophy, hearing loss, nutritional problems	Intramedullary bone, sandwich vertebra, generalized increase in bone density	7/9
CLCN7 (n=4, 23.5%)	Short stature, hepatosplenomegaly, nystagmus, immunodeficiency (fever)	Generalized increase in density	3/4
TNFSF11 (RANKL) (n=2, 11.7%)	Short stature, Optic atrophy, Early mortality (due to infection)	Generalized increase in density	1/2
TNFRSF11A (RANK) (n=1, 5.8%)	History of a fracture, hepatosplenomegaly, hearing loss	Erlenmeyer flask deformity	1/1
CA2 (n=1, 5.8%)	Pervasive developmental disorder, severe hearing loss, history of fractures	Metaphyseal widening and increased bone density	1/1

TCIRG1, T-cell immune regulator 1; CLCN7, chloride voltage-gated channel 7; TNFSF11, tumor necrosis factor ligand superfamily member 11, also known as receptor activator of nuclear factor kappa-B ligand (RANKL); TNFRSF11A, tumor necrosis factor receptor.

**Table 2B:** Biochemical, radiological, and treatment characteristics of patients with osteopetrosis.

Patient	Mutation	Ca (mg/dL) (8.5-10.5)	P (mg/dL) (3.1-6)	ALP (U/L) (280-835)	PTH (pg/mL) (15-65)	Vitamin D (ng/mL)	Radiology	Treatment	Transplantation
1	TCIRG1	8.4	2.73	445	280	9.6		Ca + vit D	+
2	TCIRG1	7.61	2.04	874	31.25	9		Ca + P + vit D	+
3	TCIRG1	7.33	2.62	825	227	32	Rickets findings (+)	Ca + P + calcitriol + pamidronate	+
4	TCIRG1	7.43	1.75	394	19.10	34.9	Rickets findings (+)	Ca + P + calcitriol	+
5	TCIRG1	8.99	3.19	663	2.68	37		Pamidronate	+
6	TCIRG1	10.5	5.33	583	3.87	67.3		Pamidronate	+
7	TCIRG1	7.02	2.48	477	362	57.9		Ca + P + calcitriol	+
8	TCIRG1	8.2	1.96	887	237	34.1		Ca + calcitriol + vit D + P	+
9	TCIRG1	6.79	2.76	294	–	21.3		Ca + P + calcitriol	+
10	CLCN7	6.76	4.98	531	347	11.9		Ca + calcitriol	+
11	CLCN7	10	6.46	363	40	32		–	
12	CLCN7	8.4	3.65	157	46.1	9.92	Rickets findings (+)	Ca + vit D	+
13	CLCN7	8.1	3.3	1294	41.2	14.4		Ca+ Vit D	+
14	TNFSF11	8.73	4.55	60	99.5	13.2		–	
15	TNFSF11	6.88	2.02	986	312.6	49	Rickets findings (+)	Ca + P + calcitriol	
16	TNFRSF11A	9.06	3,1	71	7.41	12.8		Vit D + pamidronate + denosumab	+
17	CA2	9.35	5.7	147	36.95	13.34	Rickets findings (+)	Vit D	

Ca; calcium; P, phosphorus; ALP, alkaline phosphatase; PTH, parathyroid hormone.

At the time of diagnosis, 13 patients (76.4%) had short stature (<- 2 SD). Hepatosplenomegaly was detected in 7 patients (41.1%) during the physical examination. Hepatosplenomegaly was particularly prevalent in patients with *TCIRG1* and *CLCN7* mutations. While a history of fractures was present in a total of four patients, an old fracture line was incidentally detected in one patient during radiological examination.

Ophthalmological findings such as optic atrophy, nystagmus, or strabismus were present in 10 patients (58.8%); hearing loss was present in 7 patients (41.1%). Peripheral facial paralysis was detected in one patient (*TCIRG1*) (**Table 2**).

### 3.2 Genetic distribution

Genetic analysis revealed that the most common mutation was in *TCIRG1* (n=9, 52.9%). Other mutations included *CLCN7* (n=4, 23.5%), *TNFSF11* (n=2, 11.7%), *TNFRSF11A* (n=1, 5.8%), and *CA2* (n=1, 5.8%) mutations (**Table 2**).

### 3.3 Biochemical and radiological evaluation

A total of 13 out of 17 patients (76.4%) presented with osteopetrorickets. The biochemical abnormalities in this group were in order: hypocalcemia in 11 patients, hypophosphatemia in 8 patients (all accompanied by hypocalcemia), and vitamin D deficiency in 4 patients (**Table 2**).

Patients with osteopetrorickets were compared with those without rickets in terms of age at diagnosis, genotype, calcium–phosphate abnormalities, vitamin D status, neurological findings, and transplantation status. Because of the small number of patients in the non-rickets group, the comparison was interpreted cautiously.

**Figure 1:**
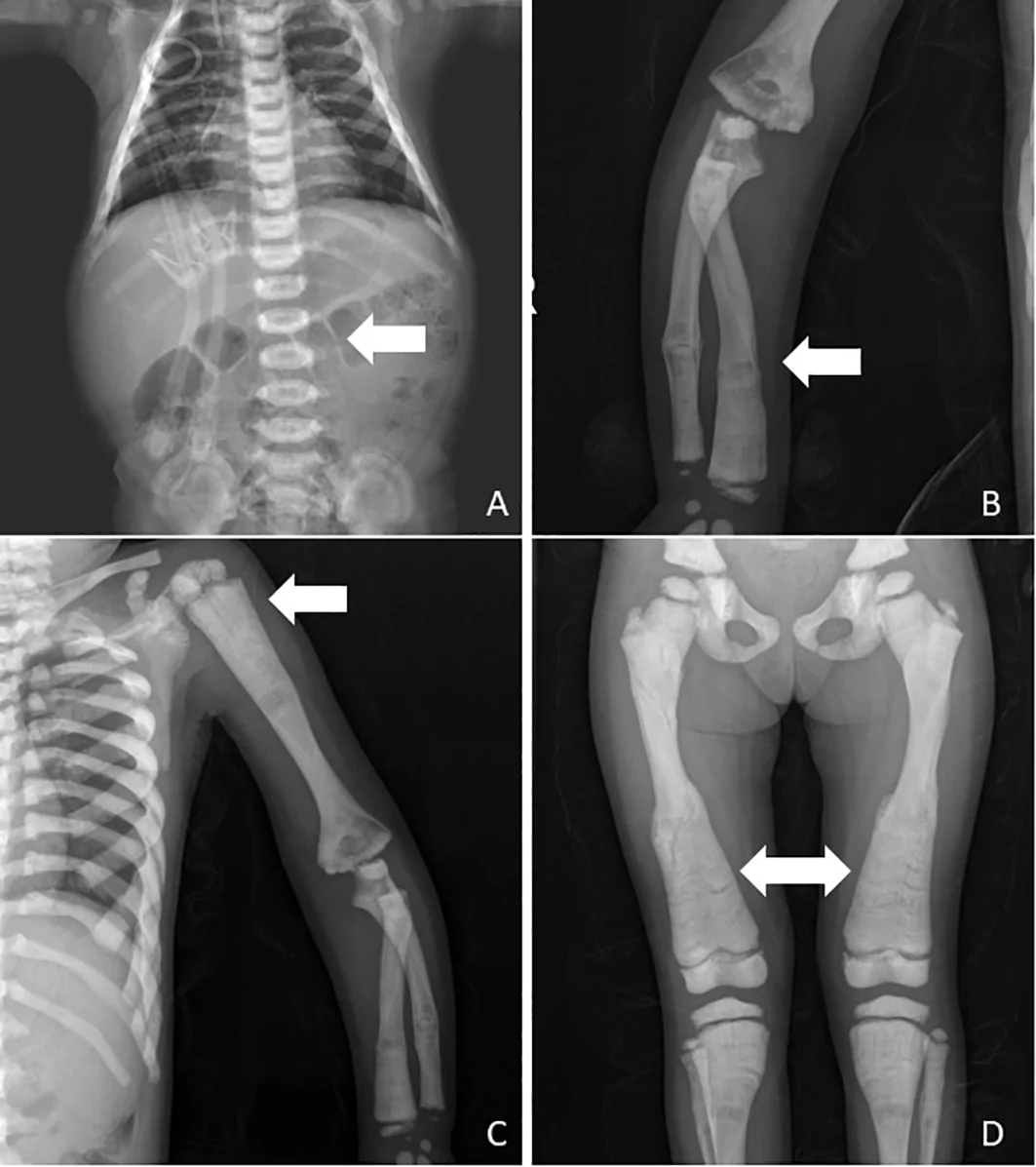
Representative radiographic findings of osteopetrosis. Panel **(A)** shows generalized osteosclerosis with possible vertebral endplate sclerosis, panel **(B)** shows fracture-related callus formation, panel **(C)** shows metaphyseal widening, and panel **(D)** hows Erlenmeyer flask deformity.

Generalized increased bone density was observed on conventional radiographs in all patients. Classic osteopetrosis-associated findings, including bone-in-bone appearance (*TCIRG1*, n=2), Erlenmeyer flask deformity (*TNFRSF11A*, n=1), and sandwich vertebrae (*TCIRG1*, n=1), were identified in a subset of patients. Pathological fractures or radiographic evidence of previous fractures were observed in patients with *TNFRSF11A* (n=1/1), *CA2* (n=1/1), and *TCIRG1* (n=2/9) mutations (**Figure 1**; **Table 2**).

Patients with hypocalcemia, hypophosphatemia, vitamin D deficiency or insufficiency, and/or radiological findings compatible with rickets were treated with calcium, calcitriol, vitamin D, and/or phosphorus supplementation according to the predefined biochemical criteria and clinical status. Urinary calcium-to-creatinine ratios were assessed in patients receiving vitamin D and/or calcium therapy; the urine test results for all patients receiving treatment were normal during follow-up.

### 3.4 Treatment and bone marrow transplant management

HSCT was performed in 11 of 13 patients (84.6%) diagnosed with osteopetrorickets. The median post-transplant follow-up period was 15 months (15 days to 5.8 years). The causes of death were interstitial pneumonia in one patient, veno-occlusive disease in one patient, sepsis in one patient, and aspiration pneumonia in one patient. Among patients with osteopetrorickets who underwent HSCT (n=11), complete clinical and laboratory recovery from rickets was achieved in seven surviving patients (7/11, 63.6%), and replacement therapies were discontinued within 15 days to 8 months.

Two patients with incompatible genetic subtypes (*TNFSF11*) or severe neurological involvement (*CA2*) received only supportive care.

### 3.5 Development of post-transplant hypercalcemia

Hypercalcemia developed in 4 of 13 transplant recipients (30.8%). Among the patients who received pre-transplant supplementation due to osteopetrorickets, hypercalcemia developed in two patients. In these cases, bisphosphonate and/or denosumab treatment was required to control hypercalcemia. Post-transplant hypercalcemia was also observed in two patients who had normal calcium metabolism before transplantation and were not receiving supplementation. These patients were managed with hydration, furosemide, and bisphosphonate therapy (**Table 3**).

**Table 3:** Clinical, biochemical, and treatment characteristics of patients who developed post-transplant hypercalcemia.

Case	Genotype	Age at HSCT	rickets	Treatment received prior to the HSCT	The time at which Ca levels begin to rise after HSCT	Max Ca (mg/dl)	P (mg/dl)	PTH (pg/ml)	Clinical symptoms	Nephrocalcinosis status	Treatment	The time Ca returns to normal
3	TCIRG1	14 mo	present	Ca+P Calcitriol	3 mo	16,13	2,62	none	absent	absent	Pamidronate	3 d
5*	TCIRG1	10 mo	absent	None	2 mo	14	5,39	none	absent	absent	Pamidronate	4 d 3 mo
6	TCIRG1	17 mo	absent	None	3 mo	12.45	5,94	5,62	absent	absent	Pamidronate	2 d
16*	TNFRSF11A	131 mo	present	Vitamin D	20 d 2 mo 3 mo	13	2,94	8,28	absent	absent	Pamidronate Denosumab	12 d 7 d 12 d

*recurrent hypercalcemia, mo, months; HSCT, hematopoietic stem cell transplantation; Ca, calcium; P, phosphorus; PTH, parathyroid hormone; mo, months; d, day.

Among the four patients who developed hypercalcemia after transplantation, two had osteopetrorickets. These patients were 14 and 131 months old at the time of transplantation and carried pathogenic variants in *TCIRG1* and *TNFRSF11A*, respectively. Hypercalcemia occurred on post-transplant day 20 in one patient and at 3 months in the other. Both patients had low age-adjusted serum phosphorus levels of 2.62 and 2.94 mg/dL, respectively.

The remaining two patients did not have rickets. Both carried *TCIRG1* mutations and underwent transplantation at 10 and 17 months of age. In these patients, hypercalcemia developed at 2 and 3 months after transplantation, respectively. Unlike the patients with osteopetrorickets, serum phosphorus levels were within the normal age-adjusted range.

Parathyroid hormone levels were unavailable in two patients, whereas the remaining two had low/suppressed PTH levels. None of the patients had documented hypercalcemia-related clinical symptoms or nephrocalcinosis. The time to resolution of hypercalcemia after treatment ranged from 3 days to 3 months. One patient with osteopetrorickets required denosumab because of recurrent/persistent hypercalcemia despite bisphosphonate therapy, after which serum calcium levels normalized.

Compared with normocalcemic transplant recipients, patients who developed hypercalcemia were descriptively evaluated according to age at HSCT, genotype, presence of osteopetrorickets, pre-transplant supplementation status, phosphorus levels, and post-transplant clinical course. Because of the small number of patients, comparative analyses were considered exploratory. No definitive causal or predictive conclusions were drawn.

## 4 Discussion

Osteopetrosis is a heterogeneous disorder resulting from a rare defect in osteoclast differentiation. Impaired bone resorption leads to a pathological increase in bone density, fractures, and neurological findings resulting from extramedullary hematopoiesis and nerve compression. In IMO, the most severe form, the maximum life expectancy without treatment is approximately ten years (21). In our study, we examined clinical and laboratory data from 17 patients across a broad genetic spectrum (*TCIRG1, CLCN7, TNFSF11, TNFRSF11A, CA2*) to assess genotype-phenotype correlations. In particular, we evaluated the course of cases presenting with osteopetrosis and changes in calcium homeostasis following HSCT.

Rickets is a well-known complication of osteopetrosis, and its pathogenesis is based on disruption of calcium-phosphorus homeostasis caused by osteoclast dysfunction. Insufficient bone resorption by osteoclasts leads to chronic hypocalcemia in the extracellular fluid. This condition causes secondary hyperparathyroidism. Elevated PTH induces hypophosphatemia by promoting renal phosphate wasting through downregulation of cotransporters in the proximal tubule. Consequently, a clinical picture of osteopetrosis-rickets emerges, characterized by severe impairment of bone mineralization. Although bone density is increased in osteopetrosis, osteopetrosis-rickets was diagnosed in 75% of the patients in our study. Hypocalcemia was observed in 64.7% of our cases, and hypophosphatemia in 47%. These findings are consistent with the observation by Wu et al. that the absence of osteoclastic activity leads to a failure to maintain serum calcium levels (15). It was noted that vitamin D deficiency (23.5%) exacerbates this condition and accentuates radiological rickets findings (cylindercell formation, widening) (15, 22).

In osteopetrosis, genetic diagnosis is important both for determining the prognosis and for establishing the indication for bone marrow transplantation. In this study, the most frequently identified mutation was *TCIRG1*, accounting for 52.9%. This mutation, which causes the most severe form—infantile malignant osteopetrosis—represents the most described form in the literature, and HSCT has been the primary treatment option for these patients (9). However, in our *TNFSF11* (*RANKL*) deficiency cases, HSCT is not curative because the defect is osteoblastic in origin; these patients received only supportive care in accordance with literature recommendations (23). The neurological findings and concomitant renal tubular acidosis observed in our case with a *CA2* mutation have once again confirmed the limited efficacy of HSCT in this genotype (24).

HSCT is currently the only effective treatment for osteopetrosis, but its efficacy depends on the patient’s genotype. It initiates bone remodeling by restoring osteoclast function (15). To the best of our knowledge, the positive effects of stem cell transplantation on intrinsic osteoclast abnormalities, abnormal bone metabolism, and extramedullary hematopoiesis are more pronounced in ARO patients harboring *TCIRG1* variants (25). However, numerous post-transplant complications have been reported in ARO patients (25). A clinically significant endocrine complication is the risk of post-transplant “rebound” hypercalcemia (26). In our study, hypercalcemia developed in 36.3% of transplant recipients. Among the 10 patients with osteopetrosis who started treatment and underwent successful transplantation, 20% (n=2) developed post-transplant hypercalcemia. Notably, post-transplant hypercalcemia developed in all patients (100%) who had normal calcium metabolism prior to transplantation. This may be related to the rapid resorption of calcium stored in bones over a long period by newly formed, healthy osteoclasts (4). The use of bisphosphonates and denosumab is recommended in the literature for the management of this condition. In our study, these agents were also successfully used in cases of resistance (5).

In osteopetrosis, cranial nerve compression, particularly optic atrophy and hearing loss resulting from narrowing of the foramina, constitutes significant neurological complications. Ophthalmological findings were observed in 58.8% of our series. This was attributed to a delay in diagnosis (median 12 months). As noted by Gelfer et al., performing HSCT after neurological damage has developed cannot reverse existing nerve damage, even if it corrects the bony defect (6).

Owing to the retrospective design and small sample size, subgroup comparisons should be interpreted cautiously, and the findings should be considered hypothesis-generating rather than definitive.

## 5 Conclusion

Osteopetrosis-related rickets is quite common in patients diagnosed with osteopetrosis. Treatment of this condition, which causes severe calcium and phosphorus imbalances, is of vital importance before transplantation. Identifying the genetic subtype prevents incorrect treatment approaches (e.g., performing HSCT on *TNFSF11*. Given the risk of severe hypercalcemia that may develop post-transplant, patients with normal pre-transplant calcium levels should be monitored more closely.

## Data Availability

The raw data supporting the conclusions of this article will be made available by the authors, without undue reservation.
